# Indirect Detection of Swine Influenza Activity in Porcine Blood Using Raman Spectroscopy and Machine Learning

**DOI:** 10.1002/jbio.202400575

**Published:** 2025-05-13

**Authors:** Aidan Paul Holman, Axell Rodriguez, Ragd Elsaigh, Roa Elsaigh, Joseph Wilson, Matt H. Cohran, Dmitry Kurouski

**Affiliations:** ^1^ Department of Biochemistry and Biophysics Texas A&M University College Station Texas USA; ^2^ Interdisciplinary Faculty of Toxicology Texas A&M University College Station Texas USA; ^3^ Nanomedicine College of Science Program Northeastern University Boston Massachusetts USA; ^4^ Cross‐Border Threat Screening and Supply Chain Defense Texas A&M University College Station Texas USA

**Keywords:** blood, influenza, machine learning, Raman spectroscopy, swine flu

## Abstract

Over the past decade, several swine influenza variants, including H1N1 and H1N2, have been periodically detected in swine. Raman spectroscopy (RS) offers a non‐destructive, label‐free, and rapid method for detecting pathogens by analyzing molecular vibrations to capture biochemical changes in samples. In this study, we examined blood serum from swine under different conditions: healthy, unvaccinated, or vaccinated against porcine reproductive and respiratory syndrome, and vaccinated swine infected with H1N1 and H1N2 variants of swine influenza. Our findings demonstrate that RS, when combined with machine learning algorithms such as partial least squares discriminant analysis and eXtreme gradient boosting discriminant analysis, can achieve accuracy rates of up to 97.8% in identifying the infection status and specific variant within porcine blood serum. This research highlights RS as a useful, novel tool for the detection of influenza variants in swine, significantly enhancing surveillance efforts by identifying animal health threats.

## Graphical abstract

Blood serum from healthy swine, unvaccinated or vaccinated against porcine reproductive and respiratory syndrome, and vaccinated swine infected with H1N1 and H1N2 variants of swine influenza was analyzed using Raman spectroscopy. Partial least squares discriminant analysis and eXtreme gradient boosting discriminant analysis of the acquired spectra enabled 97.8% in identifying the infection status and specific variant within porcine blood serum.

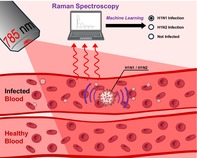

## 1 Introduction

Influenza viruses are classified into subtypes based on the antigenicity of the surface viral glycoproteins, hemagglutinin (HA) and neuraminidase (NA). In swine, three endemic subtypes predominate: swH1N1, swH1N2, and swH3N2, which have roughly equal detections over the last few years [1]. HA and NA are important determinants of virus infectivity, transmissibility, pathogenicity, and host specificity and evolve seasonally due to antigenic drift [2]. Current monitoring and diagnostic techniques, such as polymerase chain reaction (PCR)‐based assays and serological assays, are resource‐intensive and time‐consuming. For example, detection using cell cultures can take 2–7 days for sensitivities between 82% and 100%, whereas PCR‐based assays like real‐time reverse transcription PCR (rRT‐PCR) take 15 min to several hours with sensitivities between 66% and 100% [3, 4]. This raises the question of whether more rapid, highly accurate methods could be employed in situations that demand a swift response.

Raman spectroscopy (RS) is a technique that analyzes inelastically scattered light from illuminated samples and has recently demonstrated potential in identifying pathogens and their various strains by examining overall biochemical changes in the tissues they inhabit [5, 6, 7, 8, 9, 10]. For instance, Khan et al. [8] investigated biochemical alterations related to dengue infections by comparing spectra from 40 infected individuals and 25 healthy controls. They identified distinct Raman peaks that differentiated between infected and uninfected samples. Later that year, the same team used support vector machine (SVM) analysis to achieve an average accuracy of approximately 85% in distinguishing 31 dengue‐positive serum samples from 53 negative samples [6]. Senger et al. [11] applied RS to differentiate between healthy individuals and those with Lyme disease (LD) using urine samples, achieving nearly 89% accuracy through principal component discriminant analysis (PCDA). Additionally, Goff et al. [7] used RS to analyze blood samples for identifying healthy individuals versus those with three different strains of LD. They achieved 96% accuracy with mouse blood and 88% accuracy with human blood using a prebuilt partial least squares discriminant analysis (PLS‐DA) model from another similar study by Farber et al. [5]. All of these studies use RS as a non‐destructive, label‐free, and high‐throughput tool for the analysis of infection status in tissues. Giuseppe et al. [12] demonstrated that RS was capable of differentiating between healthy and Leishmania‐infected dogs, whereas Vyas et al. [13] showed that changes in the chemical composition of saliva could be used to diagnose Sjögren's disease. It should be noted that RS‐based diagnostics of disease is not limited to animals and humans. Farber et al. [14, 15] found that RS could be used to detect viral diseases in wheat and roses, while Yetru et al. [16] demonstrated experimental evidence of RS‐based diagnostics of Abutilon mosaic virus in Abutilon sp. All of these studies use RS as a non‐destructive, label‐free, and high‐throughput tool for the analysis of infection status in plants, animals, and humans.

It should be noted that unlike regular RS, which picks up the vibrations of molecules, the Raman signal can be boosted by adding plasmonic materials that create strong electromagnetic fields. This makes the signals from molecules near the surface much stronger and easier to detect. This type of analysis is called surface‐enhanced Raman spectroscopy (SERS, also called surface‐enhanced Raman scattering). Gracie et al. [17] used silver nanoparticles (AgNPs) in a colloidal solution with a DNA aptamer attached that would bind to the fragments of bacterial DNA after λ‐exonuclease digestion. They found highly distinct spectra between *Streptococcus pneumoniae* , *Haemophilus influenzae* , and *Neisseria meningitidis* with detection limits in the pico‐molar range. Wang et al. [18] used a silicon wafer chip coated with AgNPs and modified by 4‐mercaptophenylboronic acid to capture and analyze antibiotic bacteria ( *Escherichia coli* and *Staphylococcus aureus* ) within human blood. However, studies like these require more sophisticated sample preparation, namely when preparing the plasmonic material.

This study aims to evaluate whether conventional RS can distinguish between unvaccinated healthy pigs and vaccinated (against porcine reproductive and respiratory syndrome or PRRS) pigs that are either healthy or have been inoculated with low or high doses of H1N1 or H1N2 influenza variants. PRRS virus affects porcine respiratory and reproductive systems [19]. Therefore, 80% of pigs receive PRRS vaccination [20, 21, 22, 23]. We utilize PLS‐DA and eXtreme gradient boosting discriminant analysis (XGBDA) to analyze spectral data and compare the effectiveness of these machine learning models in making accurate predictions. The impact of this research lies in its potential to advance diagnostic capabilities for influenza in swine, offering a more efficient and accurate method for identifying different infection statuses and vaccine responses.

## 2 Materials and Methods

### 2.1 Pig Treatment and Inoculation

Three‐day‐old piglets were vaccinated for Influenza. After 2 weeks, the piglets were boosted with a second dose of influenza vaccine. A day later, pigs were weaned and delivered to the research barn. Pigs were housed by litter in four rooms. All pigs were vaccinated for PRRS 14 days post weaning. (*This vaccination timeline was designed to reflect standard practices in medium‐to‐large swine herds, where over 80% population receives PRRS vaccination* [20, 21, 22, 23]). Seventeen days post‐weaning, the pigs were rehoused into their appropriate treatment room. Four rooms were utilized, with one being a negative control room and the other three representing three different treatments: H1N1 flu (A/sw/Ind/A02429505/2019) high dose, H1N1 flu (A/sw/Ind/A02429505/2019) low dose, and H1N2 (A/sw/IL/A01475495/2014). The pigs receiving challenge were challenged 21 days post weaning.

### 2.2 Blood Extraction

Approximately 1 mL of swine blood was collected via venipuncture from the jugular fossa. Once withdrawn, blood was shipped (kept frozen) to Texas A&M University where vials were immediately placed and kept at −80 °C prior to measurements.

### 2.3 Raman Spectroscopy

Raman spectra were collected using a TE‐2000 U Nikon inverted confocal microscope, equipped with a 20× objective. A solid‐state laser generated 785 nm light, while power through each sample was kept at 1.8 mW. Scattered light was collected using the same magnification and directed using a 50/50 beam splitter into an IsoPlane‐320 spectrometer (Princeton Instruments) equipped with a 600 groove/mm grating. Prior to entering the spectrometer, elastically scattered photons were blocked by a long‐pass filter (Semrock, LP03‐785RS‐25). Inelastically scattered photons were collected using a PIX‐400BR CCD (Princeton Instruments).

Samples were prepared by wrapping a glass slide in one layer of foil and depositing 50 μL of blood serum into a 1‐in. × ¼ in. rectangle onto the foil. After the blood was applied to the slide, a 30‐min waiting period was observed before the analysis commenced to slightly dry the blood for an optimal signal. Thirty spectra (s, 3180 total) from each sample (*n*, 106 total) were collected at 30 s acquisitions (one accumulation per acquisition) using 8 mW of laser power. Throughout the project, four different individuals collected spectra to minimize operator bias and variability, ensuring reproducibility.

### 2.4 Data Analysis

All spectra were trimmed from 367 cm^−1^ to 1800 cm^−1^, background‐subtracted, smoothed (second order) using a Savitzky–Golay filter, baseline‐corrected (eighth order) using automatic weighted least squares, and area‐normalized before analysis using MATLAB (as displayed). Chemometric analysis of acquired spectra was done in MATLAB equipped with PLS_Toolbox 9.0 (Eigenvector Research Inc., Manson, WA). Supporting Information Figure S1 demonstrates the effects of these processing steps on the raw spectra of one sample. If sequential preprocessing was applied, it included first derivative filtering (*n* = 2, fl = 15 pt.) and mean centering. Calibration (training), cross‐validation (internal validation), and validation (external validation) models were created for each chemometric algorithm. Validation was performed by partitioning (using Kennard‐Stone algorithm) the data so that 70% was used for calibration and cross‐validation and the remaining 30% was used for testing. Venetian blinds cross‐validation was employed using 10 data splits and one sample per blind so that the left‐out data was always 10%.

Samples were categorized into the following groups: “Control” for vaccinated, uninfected pigs (*n* = 27); “UnVaxd” for unvaccinated, uninfected pigs (*n* = 16); “LowInf” for vaccinated pigs infected with a lower dose of the H1N1 variant (*n* = 23); “HighInf” for vaccinated pigs infected with a higher dose of the H1N1 variant (*n* = 20); and “SecInf” for vaccinated pigs infected with the H1N2 variant (*n* = 20). Outliers, identified as samples' spectra with data points exceeding two standard deviations within the relative class set, were manually removed (71 spectra were removed this way). Spectra from each sample were averaged by combining every five spectra, reducing the outlier‐free total of 3109 to 623 spectra before analysis. Accuracy was calculated by averaging the true positive rate (sensitivity) and true negative rate (specificity), as done for imbalanced data sets.

In PLS‐DA, the number of latent variables (LVs) for each model was determined based on the following criteria: (1) the optimal root mean square error (RMSE) values in both calibration (RMSEC) and cross‐validation (RMSECV), (2) the inclusion of variables with estimated signal‐to‐noise ratios (SNRs) greater than 3, and (3) the formation of non‐random class sets using 1000 permutations with the random *t*‐test. The optimization results for the final models can be found in Supporting Information Figure S2. The calibration, cross‐validation, and validation confusion matrices for PLS‐DA with and without preprocessing can be found in Supporting Information Table S1.

In XGBDA, the models were generated automatically without the need for variable selection (built‐in hyperparameter tuning). The optimization results for each model are presented in Supporting Information Figure S3. The calibration, cross‐validation, and validation confusion matrices for XGBDA with and without preprocessing can be found in Supporting Information Table S2.

PLS‐DA and XGBDA were selected due to their fundamentally different approaches to distinguishing features between classes. PLS‐DA is a linear model, meaning it identifies patterns based on linear relationships between spectral features. In contrast, XGBDA is a non‐linear model, capable of capturing not only linear dependencies but also more complex relationships, including sublinear and super‐linear interactions between features. By using both models, we can determine whether class separation is primarily driven by simple linear differences in spectral peaks or if a combination of linear and non‐linear patterns provides a more accurate distinction.

## 3 Results and Discussion

**FIGURE 1:**
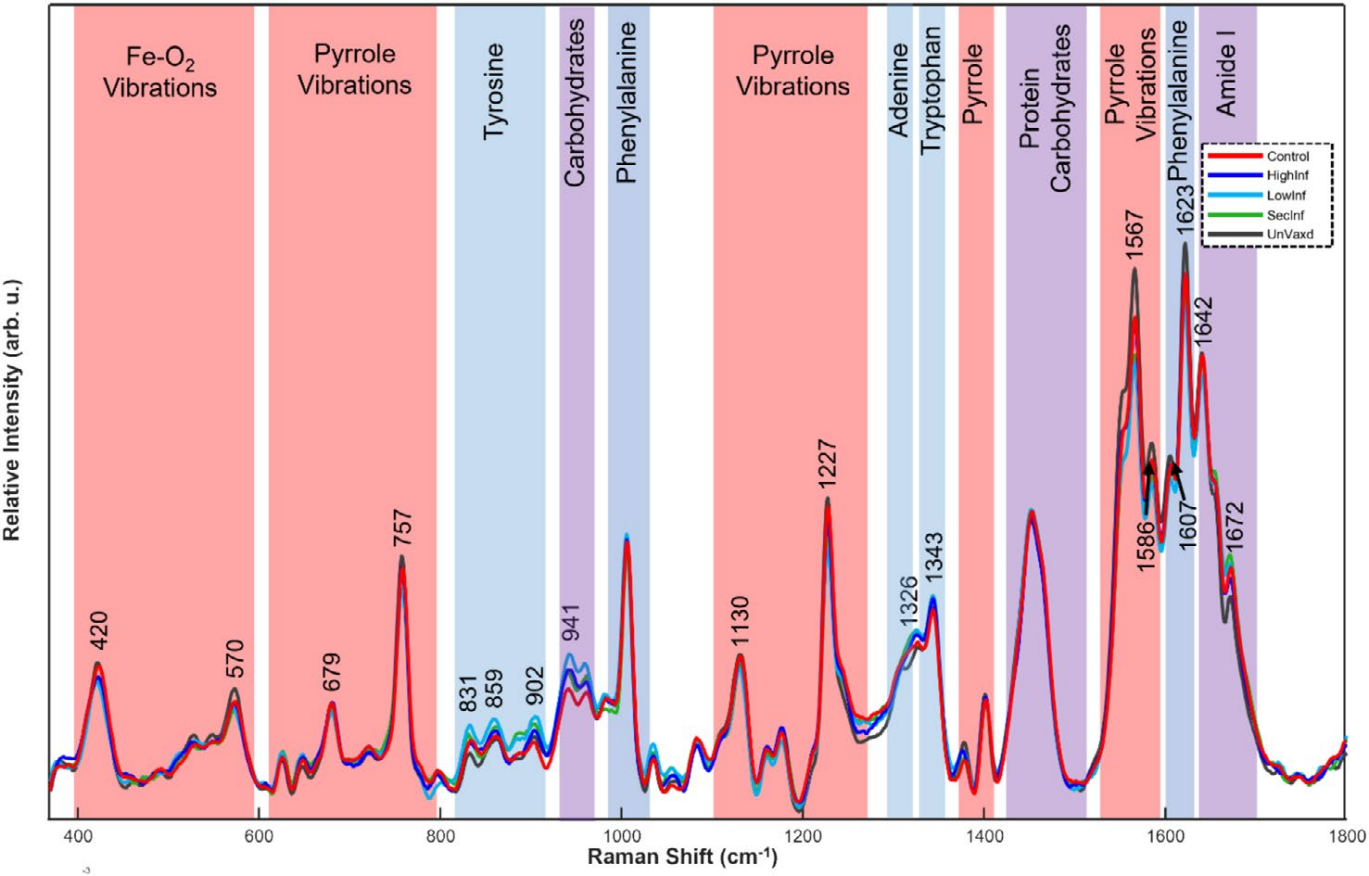
Averaged Raman spectra of all groups within the fingerprint region for blood. Highlighted Raman band ranges are assigned based on origination from heme compounds (red), aromatic amino and nucleic acids (blue), or other miscellaneous molecules (purple).

By examining the Raman spectra of blood samples, we observed subtle differences in the biochemical profiles across various groups, Figure 1 and Supporting Information Figure S4. The observed similarity across spectra is expected, as both variants of the same virus primarily affect the respiratory tract, leading to a similar immune response. Nevertheless, the Raman spectra from all groups indicate variable responses from heme compounds, aromatic amino acids, nucleic acids, and other unidentified proteins and carbohydrates.

Further statistical analysis using the Kruskal–Wallis test for analysis of variance (ANOVA) indicated that no single Raman band displayed significant mean relative intensities across all classes. However, when comparing the spectra of uninfected (Control and UnVaxd) to infected (LowInf, HighInf, and SecInf) groups, ANOVA identified 18 Raman bands with significant relative intensity differences at 420, 570, 679, 757, 831, 859, 902, 941, 1130, 1227, 1326, 1343, 1567, 1586, 1607, 1623, 1642, and 1672 cm^−1^ (specific vibrational assignments can be found in Table 1) (*p*‐values for each ANOVA test are detailed in Supporting Information Table S3).

**TABLE 1:** Significant Raman bands and their assignments.

Raman band (cm^−1^)	Assignment
420	Fe‐O_2_ bending (heme) [24]
570	Fe‐O_2_ stretching (heme) [24, 25]
679	Pyrrole symmetric bending (heme) [24, 25]
757	Pyrrole ring breathing (heme) [24, 25]
831	Tyrosine [26]
859	Tyrosine [26]
902	Tyrosine [26]
941	C‐C stretching [26]
1130	C‐C, Pyrrole ring stretching (heme) [8]
1227	C‐H, Pyrrole ring bending (heme) [24, 25]
1326	Adenine [27]
1343	Tryptophan [27]
1567	C=C, Pyrrole ring stretching (heme) [24, 25]
1586	C‐C, Pyrrole ring stretching (heme) [8, 24]
1607	Phenylalanine [26]
1623	Phenylalanine [26]
1642	C=C, Amide I [8]
1672	O‐C=O, Amide I [8]

Notably, eight of these Raman bands originate from heme compounds, and their relative intensities were significantly lower in the spectra of infected pigs. This suggests that infection with H1N1 or H1N2 in swine leads to specific biochemical changes that can be detected and quantified using RS, particularly in the heme‐related compounds. The reduced intensity in heme‐related bands may indicate a disruption in oxygen transport or other heme‐related functions in infected pigs. Zou et al. [28] reported that most down‐regulated genes following H1N1 infection were involved in oxygen transport and blood‐cytokine production. Additionally, the HA protein in H1N1 and H1N2 binds to sialic acid receptors on the surface of host cells, such as red blood cells (RBCs), facilitating viral entry, infection, and upon cell death, loss of hemoglobin [29, 30]. This could explain the lower relative intensity in heme‐related peaks from infected blood spectra compared to healthy. At the same time, influenza infections can induce systemic inflammatory responses, oxidative stress, and immune‐mediated hemolysis, which may contribute to altered RBC integrity and hemoglobin metabolism [31].

Furthermore, in 11 of the 18 significant Raman bands, infected groups exhibited lower relative intensities compared to uninfected groups. Of the seven remaining bands where infected groups showed higher relative intensities, four were associated with unknown proteins and carbohydrates. Additionally, the increase in certain protein and carbohydrate‐related bands could reflect metabolic alterations induced by the infection. This is in agreement with the plethora of studies that show H1N1 and H1N2 infection significantly lower appetite, body weight, and induce other nutrient metabolic changes [28, 32, 33, 34, 35]. These findings show that RS can be a sensitive tool in evaluating the biochemical changes involved in infections within blood.

Despite the absence of a single Raman band with consistently significant relative intensity across all five classes, machine learning algorithms can still leverage the subtle spectral variations across multiple bands to differentiate between classes. We started by using PLS‐DA without preprocessing to determine if the spectra, with only the necessary amount of filtering and normalization, are variable enough to differentiate between each other. Upon validation, the model generated an accuracy of 87% with 79.3% sensitivity and 94.7% specificity, Table 2A. When preprocessing was applied, the model generated an accuracy of 90.2% with 84.4% sensitivity and 96% specificity, Table 2B. These results suggest that RS coupled with PLS‐DA can identify between all classes with generally high accuracy, especially when first derivative filtering and mean centering are applied before analysis.

**TABLE 2:** Validation results for with and without preprocessing PLS‐DA models.

	(A) Without preprocessing (LVs = 13)			(B) With preprocessing (LVs = 13)		
	TPR/Sensitivity, %	TNR/Specificity, %	Balanced accuracy	TPR/Sensitivity, %	TNR/Specificity, %	Balanced accuracy
Control (s = 50)	80	93.382	86.691	78	95.588	86.794
LowInf (s = 49)	69.388	93.431	81.41	83.673	94.161	88.917
HighInf (s = 23)	56.522	96.933	76.728	69.565	98.773	84.169
SecInf (s = 24)	95.833	98.765	97.299	95.833	98.765	97.299
UnVaxd (s = 40)	95	91.096	93.048	95	92.466	93.733
Total	79.349	94.721	87.035	84.414	95.951	90.182

We also chose to include an additional machine learning model called XGBDA. Unlike PLS‐DA, which relies on linear assumptions, XGBDA uses decision trees to capture both linear and non‐linear relationships in the data, combining multiple trees to create a robust and flexible model. Additionally, XGBDA utilizes hyperparameter tuning, selecting the best values for a model's parameters to achieve optimal performance, whereas in PLS‐DA, models are hand‐selected by the user (albeit with existing guidelines).

We initially used XGBDA without preprocessing to replicate the approach taken with PLS‐DA and achieved an accuracy of 97.3%, with 95.7% sensitivity and 98.8% specificity, Table 3A. After applying preprocessing, the accuracy improved to 97.8%, with 96.6% sensitivity and 99% specificity, Table 3B. Importantly, preprocessing did not compromise sensitivity or specificity using both XGBDA and PLS‐DA. These results indicate that XGBDA, when coupled with RS and enhanced by first derivative filtering and mean centering, can accurately distinguish between all classes and outperforms PLS‐DA, suggesting it could be a superior model for this type of analysis.

**TABLE 3:** Validation results for with and without preprocessing XGBDA models.

	(A) Without preprocessing			(B) With preprocessing		
	TPR/Sensitivity, %	TNR/Specificity, %	Balanced accuracy	TPR/Sensitivity, %	TNR/Specificity, %	Balanced accuracy
Control (s = 50)	100	96.324	98.162	100	95.588	97.794
LowInf (s = 49)	89.796	97.810	93.803	89.796	100	94.898
HighInf (s = 23)	91.304	100	95.652	95.652	99.387	97.519
SecInf (s = 24)	100	100	100	100	100	100
UnVaxd (s = 40)	97.5	100	98.75	97.5	100	98.75
Total	95.72	98.827	97.273	96.59	98.995	97.792

Variations in sample (blood) composition and spectral noise are known to impact model bias‐variance tradeoff and thus overall robustness [36]. Regarding inter‐sample variability, we analyzed a diverse set of samples categorized into five distinct groups (Control, UnVaxd, LowInf, HighInf, and SecInf) to ensure that variations in blood composition due to infection status and vaccination were well represented. The Kennard‐Stone algorithm was used to partition the data, ensuring that 70% was used for calibration and cross‐validation, while 30% was reserved for external validation, enhancing the generalizability of the model. Furthermore, to minimize the effects of spectral noise, rigorous preprocessing steps were applied, including Savitzky–Golay smoothing, background subtraction, baseline correction (eighth order automatic weighted least squares), and area normalization. Further preprocessing, such as first derivative filtering, was used in chemometric analysis to enhance signal clarity and reduce variability caused by noise. Regarding model robustness, we employed two fundamentally different modeling approaches: PLS‐DA (a linear model) and XGBDA (a non‐linear model). This allowed us to assess whether classification was driven primarily by linear relationships in spectral features or required more complex non‐linear interactions. PLS‐DA optimization was based on RMSE values, SNR thresholds, and non‐random class set formation through 1000 permutations, while XGBDA relied on automated hyperparameter tuning. The consistency of validation results across both models (Supporting Information Tables S1 and S2) suggests that our approach effectively mitigates the influence of spectral noise and blood composition variability, supporting the robustness of our method across different populations. Thus, while inherent biological and spectral variability can impact classification models, the preprocessing strategies, cross‐validation techniques, and complementary modeling approaches implemented in this study were designed to minimize these effects and improve model reliability across diverse sample sets.

Beyond that, one might wonder how the performance and practicality of our method compares to conventional techniques, particularly in terms of sensitivity, throughput, and turnaround time (Table 4). The leading methods for identifying swH1N1 and swH1N2 are RT‐PCR and viral cultures [4]. According to the U.S. Center for Disease Control and Prevention (CDC), available, and U.S. Food and Drug Administration (FDA)‐authorized, RT‐PCR assays can take between 15 min to 5 h to complete and have sensitivities between 66% and 100% [4]. The CDC also notes that RT‐PCR assays can process up to 93 samples per turnaround time (using a 96‐well plate), resulting in throughput speeds of approximately 0.31–6.2 samples per minute [37]. In contrast, viral cultures have sensitivities between 82% and 100% for these influenza strains, with analysis times ranging from 1 to 10 days (with Shell‐vial tissue cultures being the fastest, taking 1–3 days) [3, 38]. The throughput for viral cultures is harder to quantify, as it depends on available resources such as culture media and petri dishes, as well as analyst time. However, it is generally understood that viral cultures are not used for rapid testing [4]. Our method, on the other hand, requires five spectra per sample (based on post‐acquire averaging) with 30 s between each spectrum, giving us a throughput of 0.4 samples per minute. This places our method in close proximity to the fastest RT‐PCR processing speeds. Table 4 further emphasizes that validation of our method is necessary to assess realistic turnaround times and refine the technique before it can be widely adopted.

**TABLE 4:** Comparison between turnaround time, throughput, and sensitivity of our method against current gold standards for the detection of swH1N1 and swH1N2.

Method	RS & XGBDA	RT‐PCR	Viral culture
Turnaround time	Undefined	0.25–5 h	1–10 days
Throughput (samples/min)	0.4	0.31–6.2	Undefined
Sensitivity (%)	96.6	66–100	82–100

## 4 Conclusions

The aim of this study was to evaluate the effectiveness of RS in distinguishing between various infection statuses and vaccine responses in swine, and to compare the performance of PLS‐DA and XGBDA. Our results demonstrated that RS effectively identified distinct biochemical changes associated with H1N1 and H1N2 infections, as well as distinguished between vaccinated and unvaccinated healthy individuals. XGBDA, in particular, significantly outperformed PLS‐DA, achieving higher accuracy and better performance metrics even without preprocessing. Furthermore, the application of preprocessing techniques optimized XGBDA's performance without compromising sensitivity or specificity. These findings highlight the potential of combining RS with advanced machine learning methods to enhance diagnostic capabilities, offering a robust and precise approach for identifying different influenza infection statuses in swine and potentially in broader medical contexts.

## Conflicts of Interest

The authors declare no conflicts of interest.

## Supporting information


**Figure S1.** Optimization results for the non‐preprocessed (top) and preprocessed (bottom) PLS‐DA models featuring (from left to right) (1) RMSE values between calibration (RMSEC) and cross‐validation (RMSECV), (2) estimated SNR values for each latent variable number, and (3) the results of the random *t*‐tests at 1000 permutations for each class during calibration and cross‐validation (1—Control; 2—LowInf; 3—HighInf, 4—SecInf, 5—UnVaxd)
**Table S1**. Confusion matrices for the calibration (C), cross‐validation (CV), and validation (V) PLS‐DA algorithms for non‐preprocessed (left) and preprocessed (right) models
**Figure S2**. Optimization results for the misclassification between the non‐preprocessed (left) and preprocessed (right) XGBDA models featuring the number of tress (max_depth) and learning time (eta) selected (**X**)
**Table S2**. Confusion matrices for the calibration (C), cross‐validation (CV), and validation (V) XGBDA algorithms for non‐preprocessed (left) and preprocessed (right) models
**Table S3**. Kruskal–Wallis test results for Raman bands of interest. *H—higher in relative intensity; L—lower in relative intensity*
**Figure S1**. Offset mean (solid line) and standard deviations (filled areas) for each combined group.

## Data Availability

The data that support the findings of this study are available from DHS. Restrictions apply to the availability of these data, which were used under license for this study. Data are available from the author(s) with the permission of DHS.
