# Supplementary material for: Indirect Detection of Swine Influenza Activity in Porcine Blood Using Raman Spectroscopy and Machine Learning
Source: J Biophotonics. 2025 May 13;18(7):e202400575. doi: 10.1002/jbio.202400575 (PMC12245494; doi:10.1002/jbio.202400575)
Supplement: Supplementary file 1 — Figure S1. Optimization results for the non‐preprocessed (top) and preprocessed (bottom) PLS‐DA models featuring (from left to right) (1) RMSE values between calibration (RMSEC) and cross‐validation (RMSECV), (2) estimated SNR values for each latent variable number, and (3) the results of the random t‐tests at 1000 permutations for each class during calibration and cross‐validation (1—Control; 2—LowInf; 3—HighInf, 4—SecInf, 5—UnVaxd) Table S1. Confusion matrices for the calibration (C), cross‐validation (CV), and validation (V) PLS‐DA algorithms for non‐preprocessed (left) and preprocessed (right) models Figure S2. Optimization results for the misclassification between the non‐preprocessed (left) and preprocessed (right) XGBDA models featuring the number of tress (max_depth) and learning time (eta) selected (X) Table S2. Confusion matrices for the calibration (C), cross‐validation (CV), and validation (V) XGBDA algorithms for non‐preprocessed (left) and preprocessed (right) models Table S3. Kruskal–Wallis test results for Raman bands of interest. H—higher in relative intensity; L—lower in relative intensity Figure S1. Offset mean (solid line) and standard deviations (filled areas) for each combined group. [file JBIO-18-e202400575-s001.docx]

Indirect Detection of Swine Influenza Activity in Porcine Blood using Raman Spectroscopy and Machine Learning

Aidan Paul Holman,^1,2†^ Axell Rodriguez,^2,3†^ Ragd Elsaigh,^2^ Roa Elsaigh,^2^ Joseph Wilson,^2^ Matt H. Cohran^4^ and Dmitry Kurouski^1,2‡^

† These authors contributed equally to this work.

‡ Corresponding author, e-mail: dkurouski@tamu.edu Tel: 979-458-3778.

ORCID

Aidan P. Holman: 0000-0003-4244-7348

Dmitry Kurouski: 0000-0002-6040-4213

Axell Rodriguez: 0009-0006-0640-9714

1. Department of Veterinary Physiology and Pharmacology, Texas A&M University, College Station, Texas 77843, United States
2. Department of Biochemistry and Biophysics, Texas A&M University, College Station, Texas 77843, United States
3. Nanomedicine College of Science Program, Northeastern University, Boston, Massachusetts 02115, United States

4. Cross-Border Threat Screening and Supply Chain Defense, College Station, Texas 77843, United States

**
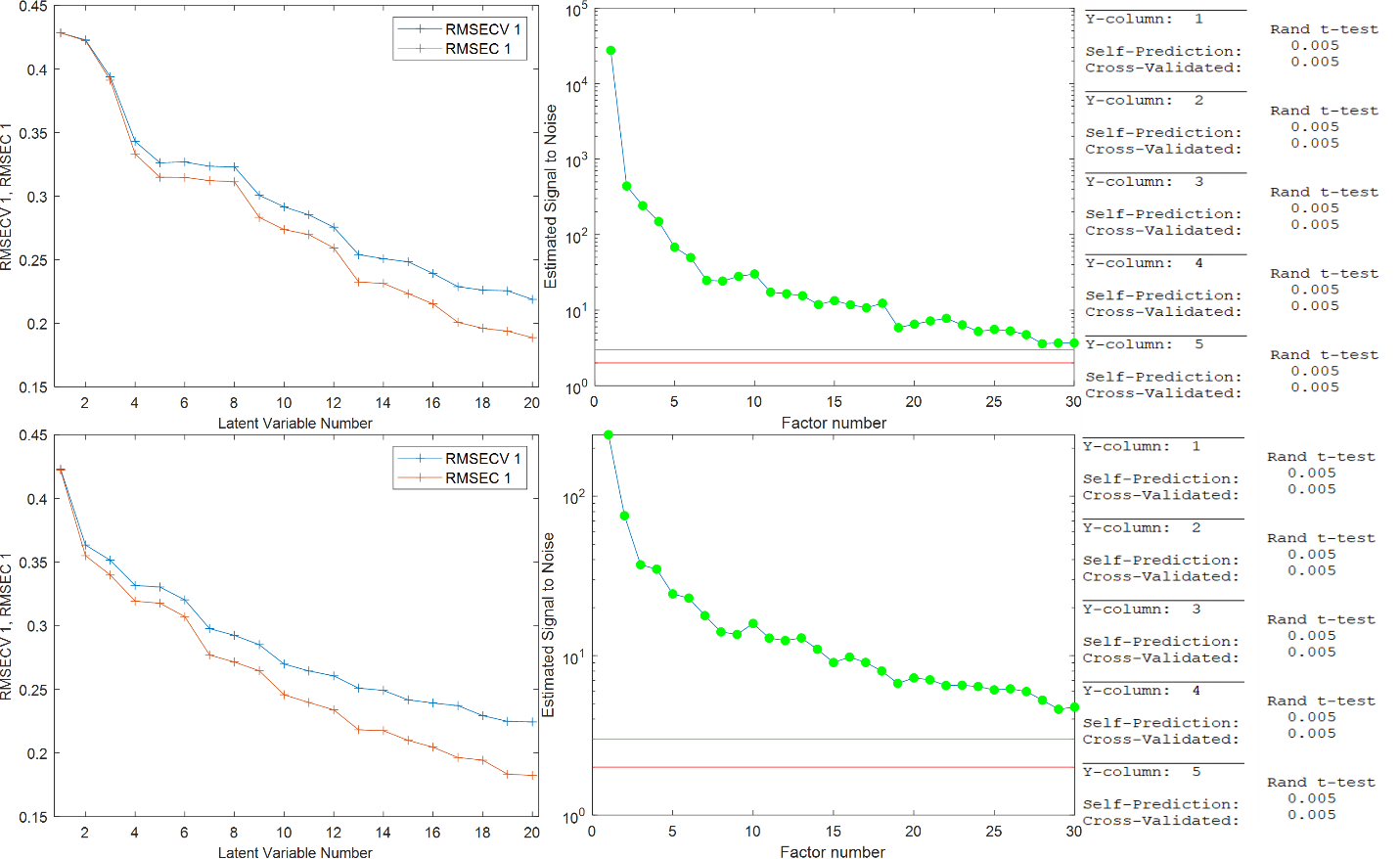
**

**Figure S1.** Optimization results for the non-preprocessed (top) and pre-processed (bottom) PLS-DA models featuring (from left to right) (1) RMSE values between calibration (RMSEC) and cross-validation (RMSECV), (2) estimated SNR values for each latent variable number, and (3) the results of the random t-tests at 1000 permutations for each class during calibration and cross-validation (1—Control; 2—LowInf; 3—HighInf, 4—SecInf, 5—UnVaxd).

**Table S1.** Confusion matrices for the calibration (C), cross-validation (CV), and validation (V) PLS-DA algorithms for non-preprocessed (left) and pre-processed (right) models.

|  | **No Preprocessing Actual Group** | | | | | **Preprocessed Actual Group** | | | | |
| --- | --- | --- | --- | --- | --- | --- | --- | --- | --- | --- |
| **Predicted as…** | **Control** (s=106) | **LowInf** (s=89) | **HighInf** (s=97) | **SecInf** (s=95) | **UnVaxd**  (s=50) | **Control** (s=106) | **LowInf** (s=89) | **HighInf** (s=97) | **SecInf** (s=95) | **UnVaxd**  (s=50) |
| Control (C) | 100 | 3 | 0 | 3 | 0 | 102 | 2 | 0 | 5 | 0 |
| LowInf (C) | 2 | 81 | 5 | 0 | 0 | 1 | 83 | 3 | 2 | 0 |
| HighInf (C) | 1 | 2 | 89 | 2 | 1 | 0 | 0 | 93 | 0 | 1 |
| SecInf  (C) | 0 | 3 | 1 | 89 | 0 | 0 | 1 | 0 | 88 | 0 |
| UnVaxd (C) | 3 | 0 | 2 | 1 | 49 | 3 | 3 | 1 | 0 | 49 |
| Control (CV) | 98 | 5 | 0 | 3 | 0 | 99 | 2 | 0 | 5 | 0 |
| LowInf (CV) | 3 | 75 | 7 | 3 | 0 | 1 | 80 | 3 | 3 | 0 |
| HighInf (CV) | 2 | 3 | 84 | 2 | 1 | 2 | 3 | 88 | 1 | 2 |
| SecInf (CV) | 0 | 3 | 0 | 85 | 0 | 0 | 3 | 1 | 86 | 0 |
| UnVaxd (CV) | 3 | 3 | 6 | 2 | 49 | 4 | 1 | 5 | 0 | 48 |
| **Predicted as…** | **Control** (s=50) | **LowInf** (s=49) | **HighInf** (s=23) | **SecInf** (s=24) | **UnVaxd**  (s=40) | **Control** (s=50) | **LowInf** (s=49) | **HighInf** (s=23) | **SecInf** (s=24) | **UnVaxd**  (s=40) |
| Control (V) | 40 | 9 | 0 | 0 | 0 | 39 | 5 | 0 | 1 | 0 |
| LowInf (V) | 1 | 34 | 8 | 0 | 0 | 1 | 41 | 7 | 0 | 0 |
| HighInf (V) | 0 | 2 | 13 | 1 | 2 | 0 | 0 | 16 | 0 | 2 |
| SecInf  (V) | 1 | 1 | 0 | 23 | 0 | 2 | 0 | 0 | 23 | 0 |
| UnVaxd (V) | 8 | 3 | 2 | 0 | 38 | 8 | 3 | 0 | 0 | 38 |


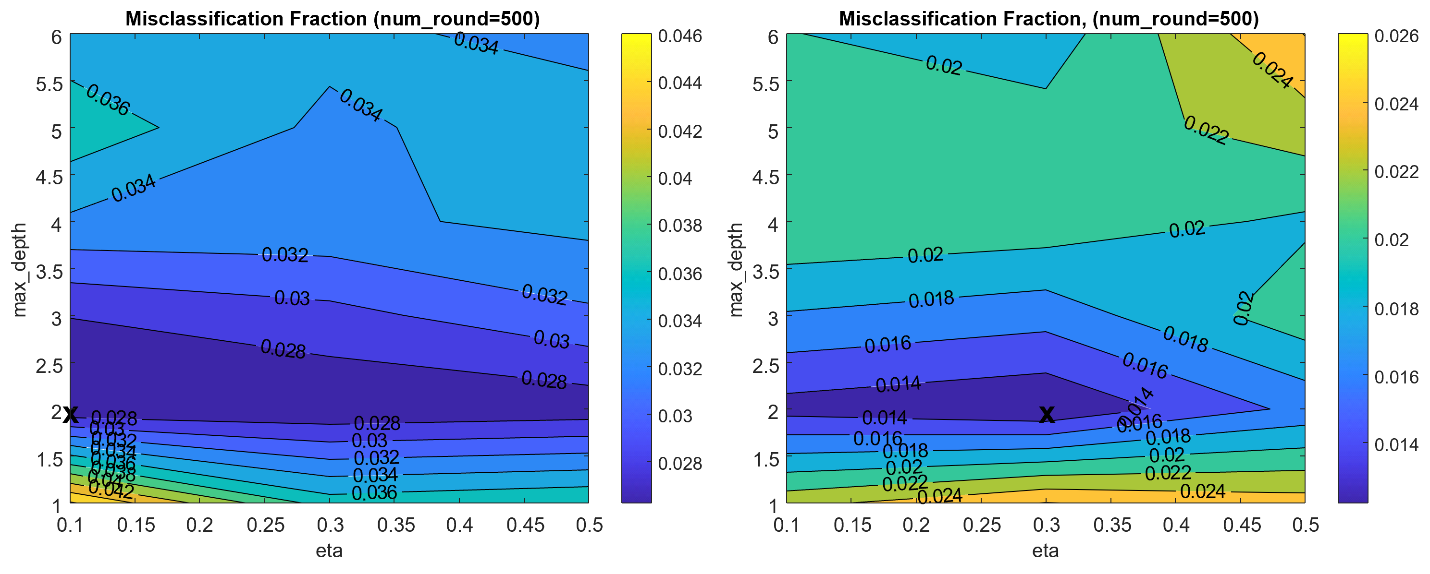


**Figure S2.** Optimization results for the misclassification between the non-preprocessed (left) and pre-processed (right) XGBDA models featuring the number of tress (max_depth) and learning time (eta) selected (**X**).

**Table S2.** Confusion matrices for the calibration (C), cross-validation (CV), and validation (V) XGBDA algorithms for non-preprocessed (left) and pre-processed (right) models.

|  | **No Preprocessing Actual Group** | | | | | **Preprocessed Actual Group** | | | | |
| --- | --- | --- | --- | --- | --- | --- | --- | --- | --- | --- |
| **Predicted as…** | **Control** (s=106) | **LowInf** (s=89) | **HighInf** (s=97) | **SecInf** (s=95) | **UnVaxd**  (s=50) | **Control** (s=106) | **LowInf** (s=89) | **HighInf** (s=97) | **SecInf** (s=95) | **UnVaxd**  (s=50) |
| Control (C) | 106 | 0 | 0 | 0 | 0 | 106 | 0 | 0 | 0 | 0 |
| LowInf (C) | 0 | 89 | 0 | 0 | 0 | 0 | 89 | 0 | 0 | 0 |
| HighInf (C) | 0 | 0 | 97 | 0 | 0 | 0 | 0 | 97 | 0 | 0 |
| SecInf  (C) | 0 | 0 | 0 | 95 | 0 | 0 | 0 | 0 | 95 | 0 |
| UnVaxd (C) | 0 | 0 | 0 | 0 | 50 | 0 | 0 | 0 | 0 | 50 |
| Control (CV) | 101 | 2 | 1 | 2 | 1 | 103 | 0 | 1 | 2 | 0 |
| LowInf (CV) | 1 | 84 | 6 | 3 | 0 | 2 | 87 | 0 | 1 | 0 |
| HighInf (CV) | 1 | 1 | 89 | 2 | 0 | 1 | 0 | 95 | 0 | 1 |
| SecInf (CV) | 0 | 1 | 0 | 88 | 2 | 0 | 2 | 0 | 91 | 1 |
| UnVaxd (CV) | 3 | 1 | 1 | 0 | 47 | 0 | 0 | 1 | 1 | 48 |
| **Predicted as…** | **Control** (s=50) | **LowInf** (s=49) | **HighInf** (s=23) | **SecInf** (s=24) | **UnVaxd**  (s=40) | **Control** (s=50) | **LowInf** (s=49) | **HighInf** (s=23) | **SecInf** (s=24) | **UnVaxd**  (s=40) |
| Control (V) | 50 | 5 | 0 | 0 | 0 | 50 | 4 | 1 | 0 | 1 |
| LowInf (V) | 0 | 44 | 2 | 0 | 1 | 0 | 44 | 0 | 0 | 0 |
| HighInf (V) | 0 | 0 | 21 | 0 | 0 | 0 | 1 | 22 | 0 | 0 |
| SecInf  (V) | 0 | 0 | 0 | 24 | 0 | 0 | 0 | 0 | 24 | 0 |
| UnVaxd (V) | 0 | 0 | 0 | 0 | 39 | 0 | 0 | 0 | 0 | 39 |

**Table S3.** Kruskal-Wallis test results for Raman bands of interest. *H—higher in relative intensity; L—lower in relative intensity.*

| **Raman Band** (cm^-1^) | **420** | **570** | **679** | **757** | **831** | **859** |
| --- | --- | --- | --- | --- | --- | --- |
| ***p*-value** | 1.37E-10 | 3.42E-10 | 2.6E-3 | 2.73E-7 | 4.73E-7 | 3.97E-6 |
| **Infected** | L | L | L | L | H | H |
| **Uninfected** | H | H | H | H | L | L |
| **Raman Band** (cm^-1^) | **902** | **941** | **1130** | **1227** | **1326** | **1343** |
| ***p*-value** | 3.32E-13 | 3.44E-12 | 2.0E-8 | 1.22E-14 | 4.04E-11 | 2.41E-12 |
| **Infected** | H | H | L | L | H | H |
| **Uninfected** | L | L | H | H | L | L |
| **Raman Band** (cm^-1^) | **1567** | **1586** | **1607** | **1623** | **1642** | **1672** |
| ***p*-value** | 4.33E-11 | 1.1E-8 | 3.78E-7 | 5.27E-11 | 5.0E-4 | 1.3E-3 |
| **Infected** | L | L | L | L | L | H |
| **Uninfected** | H | H | H | H | H | L |

**
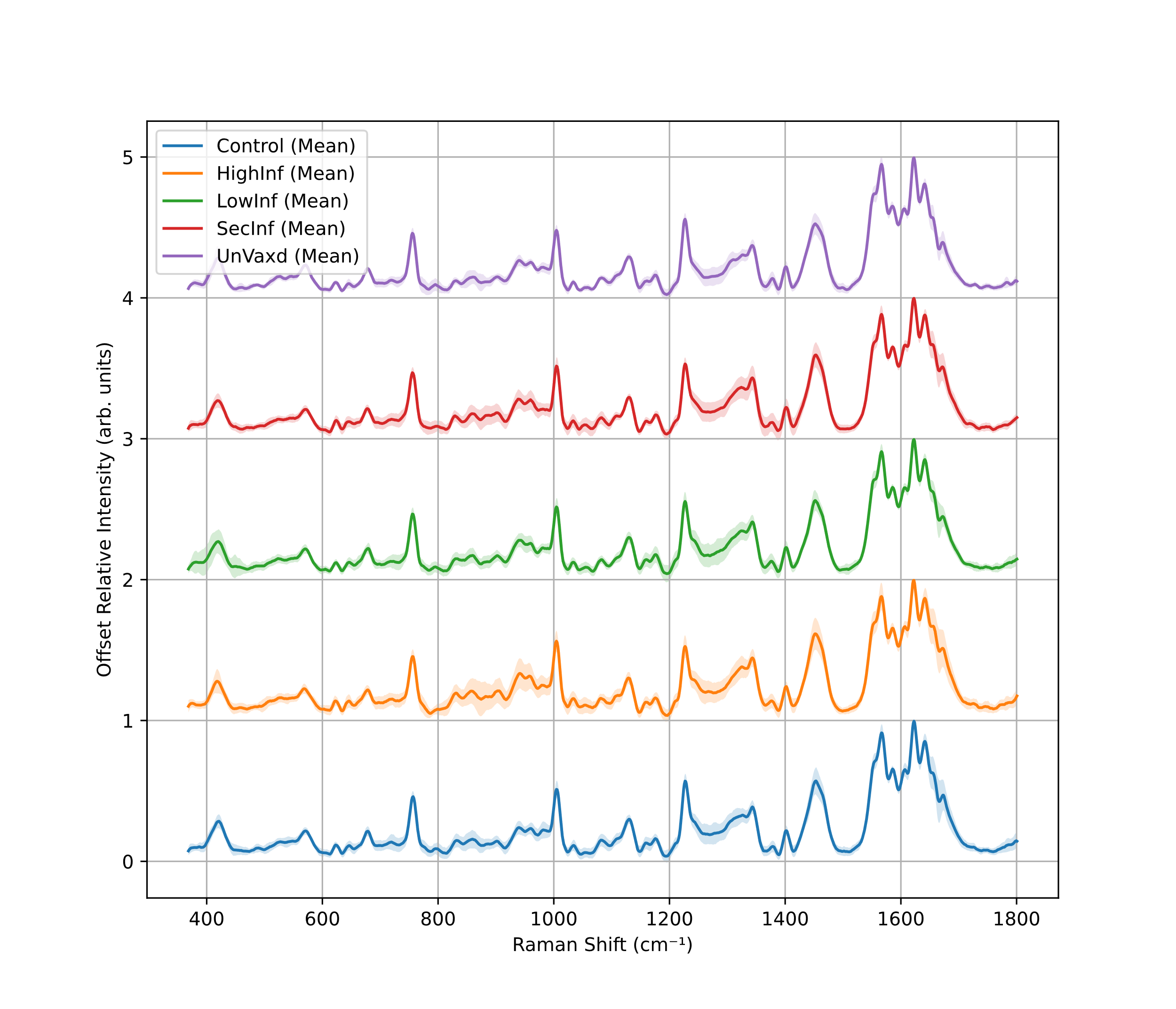
**

FIG S1:

Optimization results for the non-preprocessed (top) and pre-processed (bottom) PLS-DA models featuring (from left to right) (1) RMSE values between calibration (RMSEC) and cross-validation (RMSECV), (2) estimated SNR values for each latent variable number, and (3) the results of the random t-tests at 1000 permutations for each class during calibration and cross-validation (1—Control; 2—LowInf; 3—HighInf, 4—SecInf, 5—UnVaxd).
